# An association between *Helicobacter pylori *infection and cognitive function in children at early school age: a community-based study

**DOI:** 10.1186/1471-2431-11-43

**Published:** 2011-05-25

**Authors:** Khitam Muhsen, Asher Ornoy, Ashraf Akawi, Gershon Alpert, Dani Cohen

**Affiliations:** 1 Department of Epidemiology and Preventive Medicine, School of Public Health, Sackler Faculty of Medicine, Tel-Aviv University, Ramat Aviv, Tel Aviv, 69978, Israel; 2 Canada Israel Institute of Medical Research, Hebrew University Hadassah Medical School, Jerusalem, Israel; 3 Clalit Health Services, Shomron sub-district, Hadera, Israel

## Abstract

**Background:**

*H. pylori *infection has been linked to iron deficiency anemia, a risk factor of diminished cognitive development. The hypothesis on an association between *H. pylori *infection and cognitive function was examined in healthy children, independently of socioeconomic and nutritional factors.

**Methods:**

A community-based study was conducted among 200 children aged 6-9 years, from different socioeconomic background. *H. pylori *infection was examined by an ELISA kit for detection of *H. pylori *antigen in stool samples. Cognitive function of the children was blindly assessed using Stanford-Benit test 5^th ^edition, yielding IQ scores. Data on socioeconomic factors and nutritional covariates were collected through maternal interviews and from medical records. Multivariate linear regression analysis was performed to obtain adjusted beta coefficients.

**Results:**

*H. pylori *infection was associated with lower IQ scores only in children from a relatively higher socioeconomic community; adjusted beta coefficient -6.1 (95% CI -11.4, -0.8) (P = 0.02) for full-scale IQ score, -6.0 (95% CI -11.1, -0.2) (P = 0.04) for non-verbal IQ score and -5.7 (95% CI -10.8, -0.6) (P = 0.02) for verbal IQ score, after controlling for potential confounders.

**Conclusions:**

*H. pylori *infection might be negatively involved in cognitive development at early school age. Further studies in other populations with larger samples are needed to confirm this novel finding.

## Background

In the past few years there have been several studies, mainly from developing countries, suggesting negative influence of gastrointestinal infections in childhood on cognitive function [1,2], psychomotor development [3], and school readiness and performance [4], even when socioeconomic variables and nutritional status were controlled [1,2,4]. *Helicobacter pylori *is another microorganism acquired in early childhood that colonizes the stomach [5-8]. The prevalence of *H. pylori *infection reaches 50% by the age of five years in developing countries compared with 10%-20% in developed countries [6-8]. *H. pylori *infection is mostly asymptomatic and about 20% of infected people develop a clinical disease, usually in adulthood. *H. pylori *causes chronic gastritis, peptic ulcers and increases the risk gastric carcinoma [6,8,9]. *H. pylori *infection was also linked to depletion in iron stores in both adults and children [10-15]. It was shown that *H. pylori *infection was significantly associated with a 2.8 fold higher prevalence of iron deficiency anemia and a 1.38 fold higher prevalence of iron deficiency [13]. In a sero-epidemiologic study, *H. pylori *sero-positivity was linked to lower ferritin levels in Israeli Arab children [12]. Anemia and iron deficiency anemia were negatively correlated with cognitive development and school performance [16-19]. We therefore hypothesized that *H. pylori *infection might negatively affect cognitive development. Hypotheses on potential negative effects of *H. pylori *infection on developmental outcomes in children were raised before [20,21], however, to the best of our knowledge the association between *H. pylori *infection and cognitive development was not assessed before.

The aim of the study was to examine the association between *H. pylori *infection and cognitive development at early school age, independently of socioeconomic and nutritional factors. If this association is confirmed it would be of both clinical and public health importance.

## Methods

### Study population, setting and design

The current study focuses on a population under transition; the Israeli Arab population. This population has unique characteristics, in terms of infrastructure, health care and education systems which are similar to those existing in developed countries, while the rates of *H. pylori *infections and anemia are comparable to those reported from developing countries. The Israeli Arab population comprises 20% of the Israeli population [22]. The Israeli Arabs reside mostly in separate locations than the Jewish population, and usually in rural areas. The Israeli Arab population has lower educational levels and socioeconomic status as compared with the Jewish population [22], nevertheless this population is in positive transition, with ongoing improvement of the educational level and medical system. Israeli Arabs have mandatory health insurance according to the national health insurance law. The vaccination coverage in this population is over 95%.

This retrospective cohort study was conducted in 2007-2009, among children who participated in a previous project on *H. pylori *infection in 2004, when they were 3-5 years of age. Fifty percent of the children were *H. pylori *positive at this age [23]. Families of these children live in three villages in northern Israel. There are about 150,000 Muslim Arab inhabitants living in this region, with 3914 live births in 2007 [24]. Two of the villages have approximately 10,000 residents, and the third one is inhabited by about 14,000 residents. According to the Central Bureau of Statistics, one village belongs to cluster 2-socioeconomic status (SES), one belongs to cluster 3-SES, and the third village belongs to cluster 4-SES (for more details on the study villages see additional file 1). The clusters are on a scale of 1-10, the lower the index, the lower the SES [25]. At the national level, these villages are of low and intermediate SES levels [25], but given the variation among them, they were labeled in the present study as low, intermediate and high SES village. Drinking water supply in these villages is piped, and all households are connected to the national electricity company similarly to the rest of the country. Connection to the cable television and internet networks is also available. The educational system in these villages includes kindergartens, primary and high schools. The three villages were selected to represent different socioeconomic background within the Arab population. The characteristics of the selected villages are similar to the Israeli Arab population. For example the median age in the Israeli Arab population is 20 years [22], as compared with 18-21 years in the three villages [25]. 34% of the families in the Israeli Arab population have ≥6 persons, and 21% of the women hold a job [22], as compared with 33% and 24%, respectively in the study sample. The mean number of rooms per a household is 3.7, and the median year of schooling is 11.3-12.0 in the Israeli Arab population [22,26], as compared with 3.8 and 10 years, respectively in the study sample.

In the original study, we used cluster sampling procedure, in which 9 kindergartens (3 per village) were sampled from the kindergartens in each village. Parents of all children from each selected kindergarten were offered to participate in the study, through personal meetings at the candidates' homes.

In the current study, children born at a gestational age of 34 week or more and a birth weight of 2 kg or more were eligible to participate in the study. Among 289 participants of the 2004 study, 3 relocated their residence place, 5 could not be located, 1 child deceased due to cancer, 2 could not participate since their mothers deceased during the study period, 7 children were excluded due to birth weight of less than 2 kg or birth week less than 34. Nine additional children were excluded due to thalassemia minor (3 children), type-1 diabetes (1 child), Glucose-6-phosphate dehydrogenase deficiency with anemia (1 child), major heart defect (1 child), panhypopituitarism (1 child), hemophilia (1 child), and significant developmental delay requiring therapy (1 child). These conditions might affect cognitive function directly or might be associated with other conditions related with cognitive function e.g. hemoglobin levels. Among parents of 263 eligible children who were contacted through home visits, 41 refused to participate in the study and 222 consented, of these, 200 complied with the study procedures (i.e. compliance rate of 76%).

The Institution Review Boards of Tel Aviv University and of Hillel Yaffe Medical Center approved the study. Written informed consent was obtained from the parents' participants.

### Data collection

Information on household and socioeconomic characteristics was obtained through personal interviews held with the mothers, by trained Arabic-speakers interviewers. The questionnaire included information on age, sex, village of residence, maternal education, maternal age, paternal education, monthly family income, number of persons living in the household, and number of rooms in the household. Crowding index was calculated by dividing the number people living in a household by the number of rooms in a household.

### The outcome variable-Cognitive function

Cognitive function was measured by Intelligence Quotient (IQ) score using Stanford-Binet-5^th ^edition (*SB5*) test, performed by a trained Arabic speaking psychologist. The following parameters were assessed and reported here: full-scale IQ, non-verbal and verbal IQ. The test was performed at standard conditions, lasting on average 45 minutes. The psychologist was blinded to *H. pylori *infection status and other independent variables. The *SB5 *was scored with the *SB5 Scoring Pro*, a Windows^®^-based software program.

### Collection of stool specimens

Fresh stool specimens were obtained from children by collection cups, using the same protocol and means. After being kept and transported in cool conditions, specimens were aliquoted and frozen at the research laboratory at -70°C until tested.

### Detection of *H. pylori *infection - The independent variable

A commercial enzyme linked immunoassay kit (Premier Platinum HpSA PLUS, Meridian Bioscience, Inc., Cincinnati, Ohio) employing monoclonal anti-*H. pylori *antibody adsorbed to 96-well microtiter plates was used to detect *H. pylori *antigen in stools according to the manufacturer's instructions. Optical density values of ≥0.140 were considered positive and <0.140 were considered negative.

### Additional independent variables

#### Current hemoglobin levels

Blood collected by finger lancing was used for hemoglobin measurement employing a portable hemoglobinometer (Hemocue Hb 201+, Sweden).

#### Hemoglobin levels in early childhood

Infants in Israel are screened for iron deficiency anemia at the age 9-18 months, and the results of the participants' tests were collected from medical records.

#### Anthropometric measurements

Anthropometric measurements were performed by specially trained registered nurses. Body weight was measured to the nearest 0.1 kilogram using an analog scale (calibrated before use), and height (to the nearest 0.1 centimeter) with a stadiometer. Information on anthropometric measurements in early childhood (ages 18-30 months) was obtained from medical records. Z scores of height for age (HAZ), weight for height (WHZ), and Body Mass Index for age (BMIZ) were calculated using Epi/Info software (Center for Disease Control and Prevention, Atlanta, Georgia (CDC)). The calculations were based on the 2000 CDC growth reference curves, which were primarily based on the US National Health Examination (NHES) and the National Health and Nutrition Examination Surveys (NHANES). BMI was calculated as: weight (kg)/height (m)^2^.

#### Socioeconomic status (SES)

SES was assessed by several parameters: (1) community SES rank as classified by the Israel Central Bureau of Statistics, (2) household socioeconomic characteristics: (a) maternal education, (b) paternal education, (c) crowding index, and (d) reported family income.

In addition, a composite variable of individual level SES was created using the parameters: maternal education, paternal education, monthly family income, and crowding index. The summative scoring of this composite index was as following: each child was accredited one point if maternal education level was ≥10 years and 0 points if maternal education level was <10 years, one point if paternal education level was ≥10 years and 0 points if paternal education level was <10 years, one point if the monthly family income was >4000 New Israeli Shekels (NIS) and 0 points if the monthly family income was ≤4000 NIS, one point if the crowding index was below the median level (1.61 persons/room) and 0 if the crowding index was ≥1.61. The higher the summative score, the better the socioeconomic status. Scoring below the median level was defined as low socioeconomic status, while scoring the median level or higher was classified as high socioeconomic status.

### Statistical analysis

Differences between the villages in the independent and the outcome variables were examined using Chi square test and one way analysis of variance (ANOVA). The difference in the mean IQ levels between *H. pylori *infected children and uninfected ones was examined using Student t test. Student t test was also used to examine the difference in IQ scores in relation to sex and categorical socioeconomic characteristics. Pearson coefficients were calculated to examine the correlations between IQ levels and independent continuous variables (current hemoglobin levels, hemoglobin levels in early childhood, HAZ and WHZ scores in early childhood, and current BMIZ scores). Multiple linear regression models were used to obtain adjusted β coefficients of effect estimates, while controlling for other covariates in the models. Variables that were associated with IQ scores in the univariate analysis (P < 0.1) were included in the multivariate analysis. Additional multivariate analyses were performed, while including in the model *H. pylori *infection, the composite SES index, hemoglobin levels and current BMIZ score as a measure of nutritional status. Since socioeconomic features might affect cognitive function and given the differences in socioeconomic status among the three villages, we hypothesized that IQ scores might also differ among the villages. In addition, the three villages differed significantly in the prevalence of *H. pylori *infection, being highest in the low SES village [16]. Thus the statistical analyses were stratified by village of residence. In all analyses two tailed P < 0.05 was considered statistically significant. Data were analyzed using SPSS software (SPSS Inc, Chicago, IL) version 17.

## Results

Two hundred children (56.5% males) with a mean age of 7.8 (SD 0.84) years were included in the study. Maternal and paternal education levels were lowest in the low SES village, and more crowded households were in this village (Table 1). The prevalence of *H. pylori *infection was significantly higher among children from the low SES village than other children. The mean full-scale IQ, non-verbal IQ and verbal IQ levels of children from the low SES village were significantly lower than those of children from the intermediate and high SES villages (Table 1). These findings support our a-priori hypothesis regarding the differences between the villages in the exposure and outcome variables.

**Table 1:** Characteristics of the participants, 2007-2009

	All villages	Missing	Village SES			P value
				
	N = 200	N (%)	Low N = 83	Intermediate N = 62	High N = 55	
Maternal education ≥10 years, N (%)	100 (50.0)	-	15 (18.1)	41 (66.1)	44 (80.0)	<0.001

Paternal education ≥10 years, N (%)	98 (52.7)	14 (7.0)	22 (30.6)	38 (61.3)	38 (73.1)	<0.001

Crowding index >2, N (%)	58 (29.0)	-	47 (56.6)	9 (14.5)	2 (3.6)	<0.001

Monthly family income ≥4000 NIS, N (%)*	74 (37.9)	5 (2.5)	15 (18.3)	31 (50.0)	28 (54.9)	<0.001

*H. pylori *infection, N (%)	107 (59.1)	19 (9.5)	63 (87.5)	22 (38.6)	22 (42.3)	<0.001

Mean Full-Scale IQ (SD)	98.9 (12.6)	-	90.1 (12.0)	106.2 (8.5)	104.0 (8.9)	<0.001

Mean Non-Verbal IQ (SD)	96.6 (12.4)	-	88.4 (10.9)	103.7 (9.8)	100.9 (9.8)	<0.001

Mean Verbal IQ (SD)	101.6 (13.1)	-	92.9 (13.8)	108.4 (8.0)	107.0 (8.4)	<0.001

* NIS: New Israeli Shekel (1 NIS~3.6 US$)

### Univariate analysis

In the high SES village, the mean full-scale IQ and non-verbal IQ levels were significantly lower among children with low maternal education. *H. pylori *infected children had significantly lower full-scale IQ, non-verbal and verbal IQ scores, as compared with uninfected ones. Current hemoglobin level was significantly correlated with IQ scores (Table 2). There was no significant association between sex, paternal education, living in crowded households, hemoglobin level, HAZ and WHZ scores in early childhood, and current BMIZ score and IQ scores, neither was the composite SES index associated with IQ scores (Table 2).

**Table 2:** Univariate analysis of IQ scores correlates -high SES village ^a^

		Full-Scale IQ	Non-verbal IQ	Verbal IQ
	**N**	**Mean (SD)**	**Mean (SD)**	**Mean (SD)**

Sex				
Males	33	103.4 (9.9)	99.9 (10.7)	106.9 (9.1)
Females	22	105.0 (7.4)	102.4 (8.4)	107.2 (7.5)

Maternal education				
<10 years	11	99.5 (7.0)	94.7 (7.5)	104.4 (6.7)
≥10 years	44	105.2 (9.0)*	102.4 (9.8)**	107.7 (8.8)

Paternal education				
<10 years	14	103.7 (8.9)	99.9 (9.9)	107.5 (8.1)
≥10 years	38	104.5 (9.2)	101.6 (10.1)	107.2 (8.8)

Crowding index				
< median	22	105.9 (8.2)	102.6 (8.9)	108.8 (8.0)
≥median	33	102.8 (9.3)	99.8 (10.4)	105.8 (8.6)

Composite SES index				
Low SES	20	102.6 (8.3)	99.0 (9.3)	106.2 (7.5)
High SES	35	104.8 (9.3)	102.0 (10.1)	107.5 (9.0)

*H. pylori *infection				
Negative	30	106.3 (6.0)	103.4 (7.1)	108.9 (6.4)
Positive	22	100.5 (11.5)**	97.2 (12.5)**	103.8 (10.2) **

Hb at early childhood ^b^	51	0.14	0.21	0.04

Current Hb level ^b^	53	0.28**	0.26*	0.28

HAZ at early childhood ^b^	54	0.02	0.05	-0.02

WHZ at early childhood ^b^	54	-0.04	-0.02	-0.08

Current BMIZ ^b^	53	-0.05	-0.08	-0.01

^a ^P value were obtained by the Student t test unless otherwise is specified.

^b ^Pearson correlation.

* P < 0.1, **P < 0.05.

Hb: hemoglobin, HAZ: Height for Age Z score, WHZ: Weight for Height Z score, BMIZ: Body Mass Index Z score.

In the intermediate SES village, the mean level of full-scale IQ, non-verbal IQ and verbal IQ was significantly lower in boys than girls, and in children with low maternal and paternal education and from a lower SES (Table 3). No significant association was found between *H. pylori *infection, living in crowded households, hemoglobin levels, HAZ score in early childhood, and IQ scores. Borderline statistically significant correlations were found between current hemoglobin levels, current BMIZ score, WHZ score in early childhood and IQ parameters (Table 3).

**Table 3:** Univariate analysis of IQ scores correlates -intermediate SES village ^a^

		Full-Scale IQ	Non-Verbal IQ	Verbal IQ
	**N**	**Mean (SD)**	**Mean (SD)**	**Mean (SD)**

Sex				
Males	38	103.6 (7.2)	100.3 (7.9)	106.8 (7.1)
Females	24	110.3 (9.0)**	109.2 (10.3)***	111.0 (8.8)**

Maternal education				
<10 years	21	101.1 (7.6)	99.6 (9.5)	103.4 (6.6)
≥10 years	41	108.7 (8.0)**	105.9 (9.4)**	110.0 (7.4)***

Paternal education				
<10 years	24	102.8 (8.0)	100.5 (8.8)	105.1 (7.5)
≥10 years	38	108.4 (8.3)**	105.8 (10.0)**	110.5 (7.5)**

Crowding index				
< median	31	106.5 (9.8)	104.2 (11.2)	108.5 (8.8)
≥ median	31	106.0 (7.2)	103.3 (8.4)	108.4 (7.3)

Composite SES index				
Low SES	29	103.4 (8.4)	101.5 (10.0)	105.2 (7.6)
High SES	33	108.7 (7.9)**	105.7 (9.4)*	111.2 (7.3)***

*H. pylori *infection				
Negative	35	106.1 (9.8)	104.1 (11.0)	107.9 (9.2)
Positive	22	106.5(6.9)	102.8 (8.2)	109.8 (6.3)

Hb at early childhood ^b^	43	-0.15	-0.08	-0.22

Current Hb level ^b^	58	0.22*	0.22*	0.17

HAZ at early childhood ^b^	62	0.07	0.15	-0.01

WHZ at early childhood ^b^	62	0.22	0.22	0.17

Current BMIZ ^b^	58	0.25*	0.21	0.24*

^a ^P value were obtained by the Student t test unless otherwise is specified.

^b ^Pearson correlation.

*P < 0.1, **P < 0.05, ***P < 0.01.

Hb: hemoglobin, HAZ: Height for Age Z score, WHZ: Weight for Height Z score, BMIZ: Body Mass Index Z score.

In the low SES village, significantly lower mean levels of full-scale IQ and verbal IQ were found among children with low maternal education and those who lived in crowded households (Table 4). Neither *H. pylori *infection nor SES composite index were associated with IQ parameters. HAZ score in early childhood was significantly correlated with IQ levels. A trend of a correlation was observed between hemoglobin levels in early childhood and full-scale IQ and non-verbal IQ scores. No significant association was found between sex, paternal education, current hemoglobin levels, current BMIZ score, WHZ score in early childhood, and IQ parameters (Table 4).

**Table 4:** Univariate analysis of IQ scores correlates - low SES village ^a^

		Full-Scale IQ	Non-Verbal IQ	Verbal IQ
	**N**	**Mean (SD)**	**Mean (SD)**	**Mean (SD)**

Sex				
Males	42	89.1 (12.0)	88.0 (11.2)	91.5 (13.5)
Females	41	91.1 (12.1)	88.8 (10.8)	94.3 (14.0)

Maternal education				
<10 years	68	88.8 (11.9)	87.8 (10.1)	90.8 (13.8)
≥10 years	15	96.0 (11.2)**	91.0 (14.4)	101.6 (10.1)**

Paternal education				
<10 years	50	89.7 (13.7)	89.0 (11.6)	91.5 (15.1)
≥10 years	22	91.6 (10.4)	86.7 (14.1)	97.2 (10.4)

Crowding index				
< median	37	93.2 (12.3)	90.2 (13.0)	97.1 (13.1)
≥ median	46	87.6 (11.3)**	86.9 (8.9)	89.5 (13.5)**

Composite SES index				
Low SES	38	88.3 (12.9)	88.0 (10.7)	90.0 (14.7)
High SES	45	91.6 (11.1)	88.8 (11.3)	95.4 (12.6)*

*H. pylori *infection				
Negative	9	89.8 (11.3)	86.7 (9.6)	94.1 (14.9)
Positive	63	90.2 (12.5)	89.1 (11.4)	92.4 (14.1)

Hb at early childhood ^b^	74	0.21*	0.30**	0.14

Current Hb level ^b^	83	0.08	0.03	0.11

HAZ at early childhood ^b^	81	0.28**	0.27**	0.26**

WHZ at early childhood ^b^	80	0.04	0.05	0.04

Current BMIZ ^b^	83	0.03	0.05	0.01

^a ^P value were obtained by the Student t test unless otherwise is specified.

^b ^Pearson correlation.

*P < 0.1, **P < 0.05, ***P < 0.01.

Hb: hemoglobin, HAZ: Height for Age Z score, WHZ: Weight for Height Z score, BMIZ: Body Mass Index Z score.

### Multivariate analysis

In the high SES village, the association between *H. pylori *infection and cognitive function remained statistically significant, and the overall reduction was 6.1 points in the full-scale IQ score, 6.0 points in the non-verbal IQ score and 5.7 points in the verbal IQ score (Table 5), after controlling for maternal education, maternal age and current hemoglobin levels. In a second multivariate analysis that included *H. pylori *infection, and controlled for the composite SES index, maternal age, current hemoglobin level and current BMIZ score, *H. pylori *infection was significantly associated with 4 point lower IQ scores: adjusted β coefficient -4.1 (95% CI -6.2, -2.0) (P < 0.001) for full-scale IQ score, -4.2 (95% CI -6.5, -1.8) (P = 0.001) for non-verbal IQ score and -3.7 (95% CI -5.7, -1.7) (P < 0.001) for verbal IQ score.

**Table 5:** Multiple linear regression models of the association between *H. pylori *infection and IQ sores in Arab children, Israel

	Full-Scale IQ		Non-verbal IQ		Verbal IQ	
	**β**	**95%CI**	**β**	**95%CI**	**β**	**95%CI**

**High SES village **^a^						

*H. pylori *infection						
Negative	Reference		Reference		Reference	
Positive	-6.1**	-11.4, -0.8	-6.0**	-11.1, -0.2	-5.7**	-10.8, -0.6

Maternal education						
<10 years	-2.4	-9.1, 4.2	-5.4	-12.7, 1.8	0.9	-5.5, 7.2
≥10 years	Reference		Reference		Reference	

Current Hb level	3.5**	0.3. 6.7	3.4*	-0.1, 6.9	3.5**	0.5, 6.6

**Intermediate SES village **^b^						

*H. pylori *infection						
Negative	Reference		Reference		Reference	-1.2, 7.1
Positive	1.8	-4.2, 4.5	0.3	-4.8, 5.3	3.0	

Maternal education						
<10 years	-5.6**	-11.2, -2.2	-2.7	-8.4, 2.9	-7.9**	-12.5, -3.2
≥10 years	Reference		Reference		Reference	

Sex						
Males	-6.6**	-10.3, -1.2	-8.6**	-13.7, -3.5	-4.3**	-8.5, -0.2
Females	Reference		Reference		Reference	

Current Hb level	2.3**	0.4, 4.3	2.4**	0.2, 4.7	1.9**	0.03, 3.7

**Low SES village **^**c**^						

*H. pylori *infection						
Negative	Reference		Reference		Reference	
Positive	1.8	-6.6, 10.3	3.2	-4.4, 10.8	0.3	-9.5, 10.2

Maternal education						
<10 years	-1.4	-9.8, 7.1	0.04	-7.5, 7.6	-2.8	-12.7, 7.0
≥10 years	Reference		Reference		Reference	

Crowding index						
< median	Reference		Reference		Reference	
≥ median	-5.4*	-11.8, 0.9	-2.5	-8.2, 3.1	-7.8**	-15.2, -0.4

Hb at early childhood	4.2**	0.2, 8.2	5.2**	1.6, 8.8	3.0	-1.7, 7.7

HAZ at early childhood	4.0**	0.2, 7.8	3.3*	-0.1, 6.8	4.3*	-0.1, 8.8

*P < 0.1, **P < 0.05, Hb: hemoglobin, HAZ: Height for Age Z score.

^a ^The multivariate analysis in the high SES included the variables *H. pylori *infection, maternal education, maternal age and current hemoglobin levels. ^b ^In the intermediate SES village the adjusted model included the variables *H. pylori *infection, sex, maternal age, maternal education, and current hemoglobin level. The estimates did not changed when the variables "WHZ at early childhood" and "current BMIZ" were entered into the model. ^c ^In the low SES village the adjusted model included the variables *H. pylori *infection, maternal education, crowding index, hemoglobin levels and HAZ at early childhood.

In the intermediate SES village sex, maternal education and current hemoglobin levels were the main correlates of IQ scores, while in the low SES village, living in crowded households, HAZ score and hemoglobin levels in early childhood were the main correlates (Table 5).

## Discussion

We examined the association between *H. pylori *infection and cognitive development among school age children from different socioeconomic background. *H. pylori *infection was independently associated with a 4 to 6 point lower full-scale IQ score, as well as reduced non-verbal IQ and verbal IQ scores, in children who lived in a relatively higher SES village. To the best of our knowledge there are no published studies on the relationship of *H. pylori *infection with cognitive development.

Previous studies have shown an association between *H. pylori *infection and iron deficiency anemia [13]. In the same cohort of children, we found a 2.8 higher risk for anemia and lower mean ferritin levels at age 6-9 years in *H. pylori *infected children compared with uninfected ones, after controlling for socioeconomic confounders [27]. In a different study, *H. pylori *sero-positivity was associated with increased frequency of low ferritin levels in Arab children in Israel [12]. Iron deficiency anemia is believed to reduce cognitive abilities and school performance in children [16-19]. Lower iron stores and anemia related to *H. pylori *might in part explain the observed association between *H. pylori *infection and lower IQ scores. Another explanation may rely on the relationship between *H. pylori *infection and hypochlorhydria, which may increase the risk of diarrheal diseases resulting in malnutrition, iron deficiency anemia and eventually cognitive impairment [21]. Interestingly, *H. pylori *infection was recently linked with increased likelihood of Alzheimer disease [28] and mild cognitive impairment in older adults [29]. It was suggested that *H. pylori *eradication therapy might be beneficial to cognitive and functional status among such patients [30]. This association was explained by a cascade of events, starting with *H. pylori*-gastritis, resulting in reduced absorption of vitamin B12 and folate which lead to accumulation of homocysteine levels, which is considered a risk factor of cognitive impairment in adults [29,31].

The inverse association between *H. pylori *infection and IQ parameters was evident only in children from the higher SES village. We believe that in this homogeneous subgroup, the role of other factors such as low maternal education and nutritional status is limited and does not mask the separate effect of *H. pylori *infection on cognitive development. We cannot rule out the possibility of lacking the statistical power to detect a significant association between *H. pylori *and IQ scores in the low SES village, in which almost 88% of the children were infected with *H. pylori*.

The role of the duration of *H. pylori *infection on cognitive function was not examined in the current study, since only 140 children were examined for *H. pylori *infection at both pre-school age and school age [32]. In this cohort of children, *H. pylori *infection was mostly acquired at pre-school age; 49.3% of the children were *H. pylori *positive at both age 3-5 years and 6-9 years, and 10.0% acquired the infection between these ages [32]. Hemoglobin levels were assessed as one of the covariates in our study, and a positive correlation was found between current hemoglobin levels and IQ scores in children from the high and intermediate SES villages, while in the low SES village hemoglobin levels in early childhood correlated positively with IQ scores. Although the impact of the duration of anemia on IQ scores was not assessed, we found a significant and positive correlation between hemoglobin level at the age of 6-9 years and hemoglobin levels in early childhood (r = 0.25, P = 0.001), suggesting that current hemoglobin level is likely influenced by past hemoglobin status.

A previous study showed that stunting, a measure of protein-energy malnutrition, in the first two years of life was associated with diminished cognitive function at school age [33]. Stunting is uncommon among the studied population (1.5% by 18-30 months). Our results among children from the low SES village indicate that even when stunting is rare, the greater height for age Z score, the better is the cognitive development.

We examined the novel finding on the association between *H. pylori *infection and cognitive function while broadly controlling for household and community socioeconomic characteristics, and nutritional status by stratification and multivariate analyses. We also restricted the participation in the study to children born at a gestational age of 34 week or more and a birth weight of 2 kg or more, and excluded children with medical conditions that might be associated with developmental outcomes to avoid confounding effect of these variables. The study population, Israeli Arabs, has unique characteristics. The infrastructure, health care and education systems are similar to those existing in developed countries while the rates of *H. pylori *infections and anemia are similar to those reported from developing countries. These can be regarded as strengths of the present study. Our study has also worth mentioning limitations. First, the small sample size limited the precision of the effect estimates, and limited our ability to assess the role of the duration of *H. pylori *infection and the duration of anemia on cognitive development. Residual confounders could also be still present. At this stage, we can not draw conclusions regarding a causal association between *H. pylori *and IQ scores.

## Conclusions

Our findings indicate that *H. pylori *infection is associated with lower cognitive function at early school age, independent of socioeconomic and nutritional status, in relatively higher socioeconomic community. Further studies in other populations with larger samples are needed to confirm our results.

## Competing interests

The authors declare that they have no competing interests.

## Authors' contributions

DC and KM conceived the study and planned it. DC supervised all aspects of its implementation and KM coordinated the study and led the writing of the manuscript. AA performed the cognitive assessments and AO supervised the cognitive assessment process. GA assisted substantially in the acquisition of data. DC, KM and AO worked on the data analysis and interpretation of the findings. All authors helped to conceptualize ideas, interpret findings, and review drafts of the manuscript. All authors read and approved the final manuscript

## Pre-publication history

The pre-publication history for this paper can be accessed here:

http://www.biomedcentral.com/1471-2431/11/43/prepub

## Supplementary Material

Additional file 1 Characteristics of the three study villages, as published by the Israel Central Bureau of Statistics, 2006 Click here for file
