# Supplementary material for: An association between Helicobacter pylori infection and cognitive function in children at early school age: a community-based study
Source: BMC Pediatr. 2011 May 25;11:43. doi: 10.1186/1471-2431-11-43 (PMC3121602; doi:10.1186/1471-2431-11-43)
Supplement: Additional file 1 — Characteristics of the three study villages, as published by the Israel Central Bureau of Statistics, 2006 [file 1471-2431-11-43-S1.DOC]

|  | **Village's Socioeconomic Level** |  | **Characteristic** |
| --- | --- | --- | --- |
| **High** | **Intermediate** | **Low** |  |
| 4 | 3 | 2 | Socioeconomic cluster |
| 14430 | 10265 | 11409 | Total population |
| 21 | 21 | 18 | Median age, years |
| 30.5 | 25.9 | 41.5 | % of families with ≥ 4 children |
| 23.6 | 16.7 | 9.8 | Rate of motorization* |
| 9.4 | 5.8 | 5.6 | % of new motor vehicles** |
| 1908 | 1552 | 1300 | Average income per capita, NIS*** |
| 10.6 | 4.0 | 1.5 | % students among 20-29 year old subjects |
| 35.6 | 25.8 | 16.0 | % entitled to matriculation certificate among 17-18 year old subjects |
| 50.2 | 53.8 | 57.7 | % of sub-minimum wage earners |
| 2.8 | 1.2 | 0.3 | % of earners above twice the average wage |

**Appendix 1: Characteristics of the three villages as published by the Israel Central Bureau of Statistics, 2006**

***** Rate of motorization: (number of private cars /total population in 2006) × 100

** Number of new private cars/ number of private cars in 2006.

New cars: Produced in ≥ 2002

*** NIS: New Israeli Shekels (1 NIS~ 3.6 US$).

**Source:** Israel Central Bureau of Statistics. Available at: <http://www.cbs.gov.il/www/publications/local_authorities06/local_authorities_h.htm>. (Accessed March 6, 2011).
